# Case Report: A case of unexplained retinoschisis

**DOI:** 10.3389/fmed.2025.1546953

**Published:** 2025-09-30

**Authors:** Nan Wang, Yu Zhang, Fuxiang Yuan, Chunning Zhao, Zhanyu Zhou

**Affiliations:** Department of Ophthalmology, Qingdao Hospital, University of Health and Rehabilitation Sciences (Qingdao Municipal Hospital), Qingdao, China

**Keywords:** retinoschisis, corticosteroid, etiology, case report, inflammation

## Abstract

**Background:**

Retinoschisis is characterized by separation of the neurosensory retina into two layers. The traditional pars plana vitrectomy with internal limiting membrane peeling and macular buckling has been shown to resolve retinoschisis. We report a case of unknown cause of retinoschisis in a young adult.

**Case presentation:**

A 24-year-old man presented with an 18-day history of metamorphopsia and blurred vision in his left eye. Optical coherence tomography revealed retinoschisis in the macula, the temporal side of the macula, and near the superior and inferior vascular arches. The patient was treated with oral corticosteroids. His clinical characteristics and status of retinoschisis were monitored at each visit. The retinoschisis initially became aggravated; however, it had completely resolved by the 40-day follow-up visit.

**Conclusion:**

Clinicians should be aware that, for patients with clinically identified posterior vitreous detachment (PVD) without vitreomacular traction (VMT), follow-up observation or empirical treatment with glucocorticoids can be considered to observe the disease outcome.

## Introduction

Retinoschisis is characterized by separation of the neurosensory retina into two layers. This most commonly occurs between the inner nuclear layer and the outer plexiform layer or outer nuclear layer (1). Retinoschisis may develop in the macular and/or peripheral region. The most common etiologies include juvenile X-linked retinoschisis, degenerative myopia, vitreomacular traction (VMT) syndrome, and congenital optic disc abnormalities (2, 3). We herein report a case of unknown cause of retinoschisis in a young adult. The retinoschisis was neither inherited nor associated with myopia, VMT syndrome, pit macular syndrome, or glaucoma.

## Case presentation

A 24-year-old man presented with an 18-day history of metamorphopsia and blurred vision in his left eye. His initial best-corrected visual acuity was 20/40 in the right eye and 20/40 in the left eye. Manifest refraction was −5.5DS/−3.25 DC × 145 in the right eye and −5.0DS/−3.25 DC × 170 in the left eye. The intraocular pressure was 18 mmHg in both eyes. A review of the patient’s medical history revealed that he had undergone pupilloplasty 1 month previously because of diplopia in the left eye. He had been diagnosed with bilateral lens dislocation and Marfan syndrome at the age of 8 years; his father also had Marfan syndrome. Although the patient underwent phacoemulsification and intraocular lens implantation in both eyes, he was diagnosed with bilateral intraocular lens subluxation 5 years later. Then, he underwent vitrectomy combined with intraocular lens suspension in the left eye and intraocular lens suspension in the right eye. At 15 years of age, he underwent a binocular strabismus operation.

**Figure 1:**
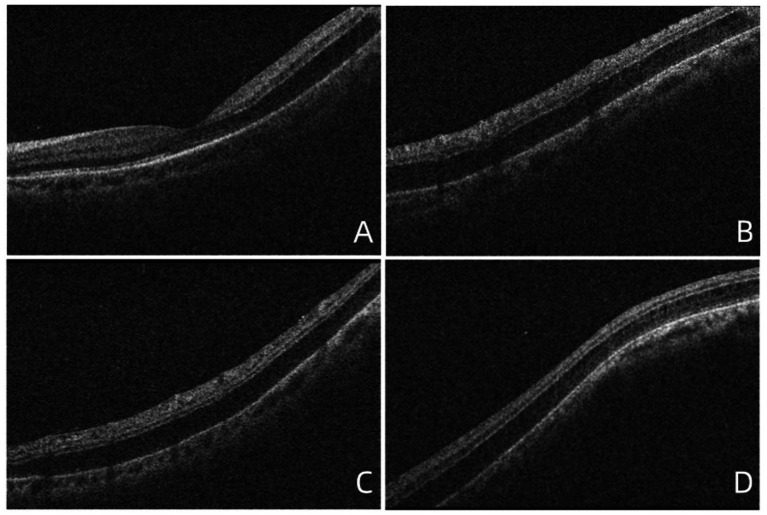
OCT images showed the retinoschisis. **(A)** The splitting of the outer plexiform layer in the macula, with a complete PVD, lack of vitreomacular traction, or epiretinal membrane. **(B)** The retinoschisis near the superior vascular arch. **(C)** The retinoschisis near the inferior vascular arch. **(D)** The retinoschisis in the temporal side of the macula.

**Figure 2:**
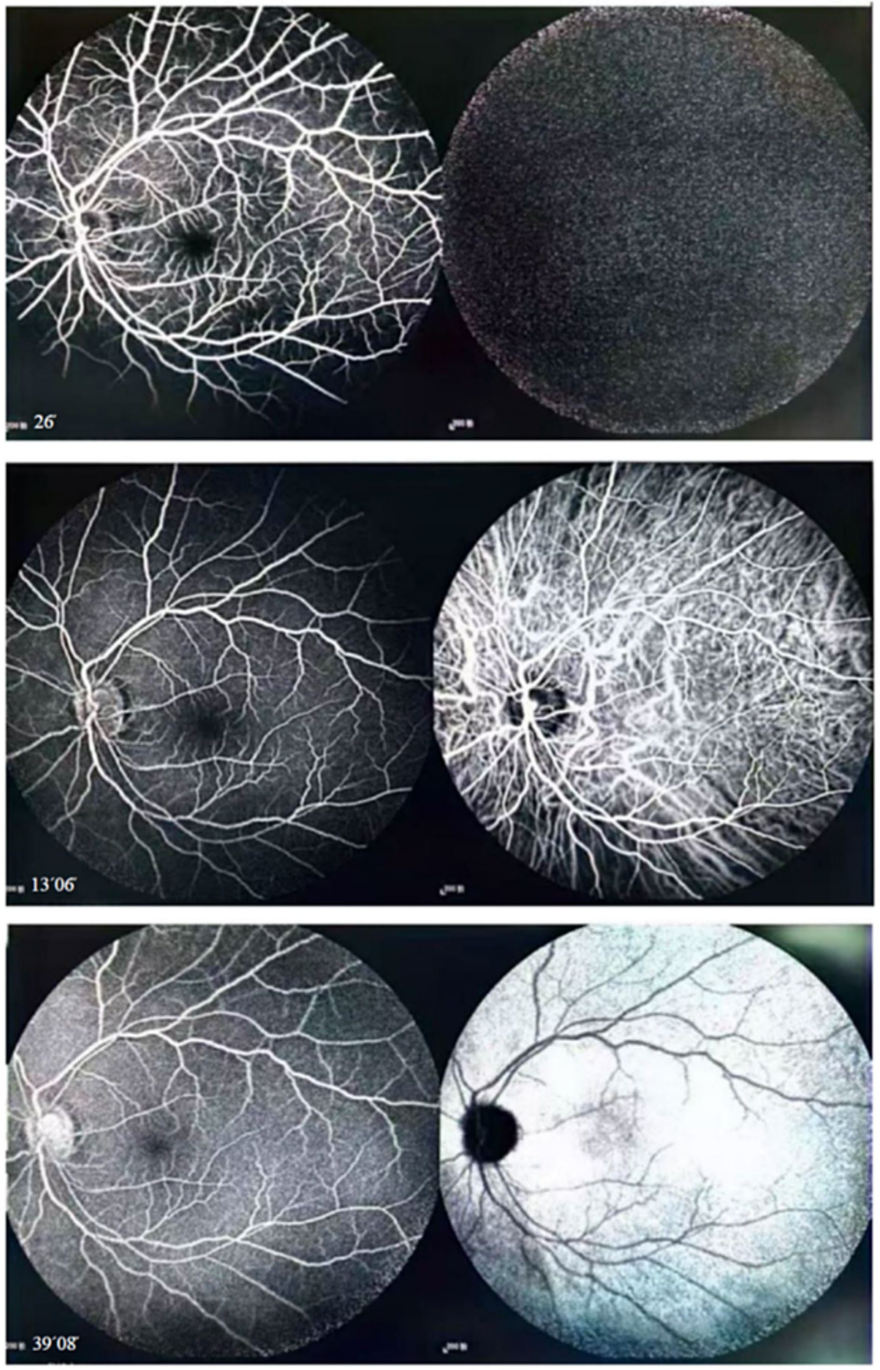
Fluorescein and indocyanine green angiography revealed no capillary leakage in the macula or optic disc at all stages.

**Figure 3:**
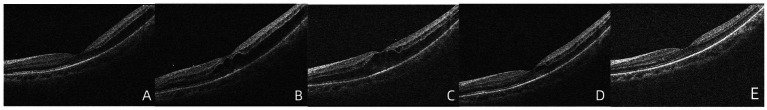
**(A,B)** OCT demonstrated that foveoschisis was aggravated 2 weeks after the patient was diagnosed and the foveoschisis cavity was expanded, and the best-corrected visual acuity (BCVA) decreased to 20/66. **(C)** Twenty-seven days after diagnosis, the OCT demonstrated that there was no obvious improvement in foveoschisis. **(D)** Forty days after diagnosis, the foveoschisis began to improve significantly; the volume of the cavity was significantly reduced. **(E)** Fifty-five days after diagnosis, the retinoschisis in the macula had resolved completely in the OCT appearance, with BCVA recovering to 20/33.

**Figure 4:**
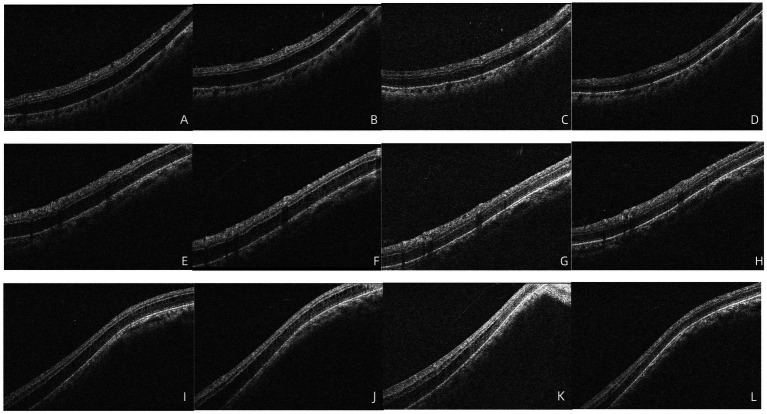
**(A–D)** OCT demonstrated that no significant improvement in retinoschisis near the inferior vascular arch 2 weeks after diagnosis, 27 days after diagnosis, the retinoschisis cavity was reduced, 40 days after diagnosis, the retinoschisis had completely resolved. **(E–H)** OCT demonstrated that no significant improvement in retinoschisis near the superior vascular arch 2 weeks after diagnosis, 27 days after diagnosis, the retinoschisis cavity was reduced, 40 days after diagnosis, the retinoschisis had completely resolved. **(I–L)** OCT demonstrated that no significant improvement in retinoschisis near the temporal side of the macula 2 weeks after diagnosis, 27 days after diagnosis, the retinoschisis cavity was reduced, 40 days after diagnosis, the retinoschisis had completely resolved.

At the time of the patient’s visit, the axial length was 25.33 mm in the right eye and 25.16 mm in the left eye; his axial length 1 month ago was 25.24 mm in the right eye and 25.14 mm in the left eye. There was no significant difference in axial length between them. The corneal curvatures of the right and left eyes were 44.00 and 43.25 D, respectively. Anterior segment examination was normal, and no inflammatory cells were observed in the anterior chamber or vitreous of the left eye. B-ultrasonography revealed no vitreoretinal traction. Optical coherence tomography (OCT) demonstrated retinal splitting in the macular area, on the temporal side of the macula, and near the superior and inferior vascular arches, occurring in the outer plexiform layer without a macular hole, VMT, or macular epiretinal membrane (ERM) (Figure 1). Fluorescein and indocyanine green angiography revealed no capillary leakage in the macula or optic disc (Figure 2). There were no obvious abnormalities in the binocular visual field. Although no active inflammation was present in the left eye, the patient was given empirical therapy including oral prednisolone at 60 mg/day for 1 week, followed by 50 mg/day for 1 week. Steroid eye drops were prescribed as well. After this 2-week treatment, OCT showed that the retinoschisis had become aggravated, and the best-corrected visual acuity had decreased to 20/66 (Figures 3A,B and Figures 4A,B,E,F,I,J).

Because the patient’s condition had not improved with treatment, he visited Beijing Tongren Ophthalmology Center and Peking Union Medical College Hospital for genetic testing. The results ruled out congenital retinoschisis. The rest of the examination results were consistent with our findings. In Beijing, the patient was given the option of either observation or posterior scleral reinforcement, and he elected observation.

The patient visited our hospital again after returning from Beijing (27 days after diagnosis). OCT demonstrated that there was no obvious improvement in the foveoschisis (Figure 3C); however, the retinoschisis near the inferior (Figure 4C), superior (Figure 4G) vascular arches and the temporal of macula (Figure 4K) had begun to improve. The prednisolone was reduced to 30 mg/day and then reduced by 10 mg every 3 days. Forty days after diagnosis, the foveoschisis began to markedly improve (Figure 3D), and the retinoschisis near the inferior (Figure 4D) and superior (Figure 4H)vascular arches and the temporal side of the macula (Figure 4L) had completely resolved. Fifty-five days after diagnosis, OCT showed that the retinoschisis in the macular region had also completely resolved, with the best-corrected visual acuity recovering to 20/33 (Figure 3E). After a follow-up observation of 14 months, the vision of his left eye was stable, and macular OCT indicated that the structure of each layer of the retina was normal.

## Discussion and conclusion

We present a case of retinoschisis that was neither inherited nor associated with VMT, optic pits, or glaucoma. Although the patient was myopic, the mechanisms of myopic foveoschisis include progressive posterior staphyloma (4, 5), increased axial length (4, 5) and continuous vitreoretinal traction caused by the posterior hyaloid (6), incomplete posterior vitreous detachment (PVD) with vitreomacular adhesion (7), or ERM formation (8). The patient had undergone vitrectomy and therefore was not at risk for VMT, which was confirmed by the OCT and ultrasound examinations. Furthermore, the retinoschisis resolved after 1 month, and the patient did not develop an increasing axial length or progressive posterior staphyloma; therefore, there was no traction force that could have caused retinal splitting. Ober et al. (9) reported that, among 22 eyes of 17 patients with retinoschisis, 16 eyes were myopic, and no eyes demonstrated features of vitreoretinal interface disorders or myopic traction maculopathy. Although possibly similar to our case, the red-free images in their study showed typical radial spoking around the fovea, and there were no significant changes in the examination findings after 6 months to 5 years of follow-up. Maruko et al. (2) reported that, among five eyes of five patients with idiopathic foveomacular retinoschisis, none of the cases were inherited or associated with VMT syndrome, optic pits, or glaucoma. However, their study focused on patients with hyperopia with short axial length. Thus, our patient had a different type of retinoschisis than those observed in these previous studies.

Because VMT, optic pits, and glaucoma were absent, we questioned whether a relationship was present between the occurrence of retinoschisis and intraocular inflammation. Some investigators have presumed that retinoschisis is a result of neovascularization, inflammatory exudates, and accumulation of fluid within the retina (10) or a result of vascular abnormalities (specifically intraretinal leakage from telangiectatic retinal vessels) rather than a result of tractional forces (11). Inflammation may result in complex cellular reactions involving a variety of cytokine-and angiogenesis-mediated retinal vascular changes, including vascular dilation and neovascularization. Retinoschisis may be the result of serous leakage from the telangiectatic retinal vessels with low-grade intraretinal edema and cyst formation. Enough intraretinal leakage can eventually result in retinal saturation and the progression to retinoschisis. The ophthalmic examination did not detect any inflammatory cells in the anterior chamber and vitreous cavity, and the fundus fluorescein angiography did not reveal any leakage in the macula and optic disc. Therefore, the occurrence of retinoschisis in the patient is not related to intraocular inflammation. However, in cases where the cause was unknown, based on clinical treatment experience, oral corticosteroids were given. During the follow-up, the retinoschisis first aggravated and then completely resolved. Did glucocorticoids actually have an effect? With this question in mind, we reviewed the relevant literature and found that corticosteroids are used to treat retinal diseases due to their anti-inflammatory properties. Corticosteroids are used in retinal diseases for their general anti-inflammatory effects, which result from the upregulation of anti-inflammatory proteins and downregulation of pro-inflammatory factors (12, 13). There are reports of patients with X-linked retinoschisis whose intraretinal or subretinal fluid and disease severity decreased following treatment with intravitreal corticosteroids (13, 14). The patient we reported showed no obvious signs of intraocular inflammation and was treated with oral corticosteroids as an empirical treatment. In addition, to the best of our knowledge, no reports have described the resolution of retinoschisis with oral corticosteroids.

Analyzing from the cellular level, Müller cells play an important role in maintaining the normal morphological structure of the retina because they span almost the whole thickness of the retina. These cells in the parafoveal region display a characteristic Z-shaped anatomical configuration (15). The horizontal part of Z-shaped Müller cells contributes to the Henle fiber layer, which is often considered a structural weak point of the retina because it is the most common site of intraretinal splitting. Govetto et al. (15) established a mathematical model of force transmission and found that traction can cause changes in the Z-shaped structure of Müller cells and disrupt the stability of the retinal interlayer structure, resulting in retinal splitting. The glial fibrillary acidic protein expressed by Müller cells plays an important role in this process (16–18). The mechanism of retinoschisis in our case was closely related to the changes in morphology and glial fibrillary acidic protein expression in Müller cells; the main difference was that our patient did not have VMT. This led us to consider the potential cause of these changes. This case was unilateral and therefore not likely to have involved a systemic factor such as an inherited condition; rather, a topical abnormality was more probable. Although no known disease-causing mutations were found, the patient may have carried unrecognized pathogenic mutations.

Is there a possibility of spontaneous resolution for the patient’s retinoschisis? Polascik et al. (19) described the case of a 65-year-old man with severe myopia of 13.00 D. Examination revealed the presence of a posterior staphyloma accompanied by macular retinoschisis and detachment. The patient was placed under conservative monitoring. During the following 18 months, his vision improved, and OCT showed a significant reduction in the size of the retinoschisis. Although the pathological mechanism of spontaneous resolution of retinoschisis was not discussed in their article, the patient had complete PVD with neither VMT nor an ERM, similar to our patient. Therefore, we consider that spontaneous resolution may have been possible because of the lack of traction on the macula.

In summary, the case we reported showed a clinical outcome of initial worsening followed by complete remission of retinoschisis. However, the underlying cause of the disease is still unclear, and it is uncertain whether glucocorticoids play a role in this process and whether there is a possibility of spontaneous resolution. Similar reports have not been seen in the past. However, the outcome of our case suggests that, in patients with clinically identified PVD without VMT, follow-up observation or empirical treatment with glucocorticoids may be considered to observe the disease progression.

## Data Availability

The original contributions presented in the study are included in the article/Supplementary material, further inquiries can be directed to the corresponding author.

## Supplementary material

SUPPLEMENTARY FIGURE 1 B-ultrasonography revealed no vitreoretinal traction of the left eye.

SUPPLEMENTARY FIGURE 2 Two weeks after the patient was diagnosed, although the OCT demonstrated that the retinoschisis was aggravated, there was no obvious abnormality in the binocular visual field.
