# Supplementary figures and images for: Case Report: A case of unexplained retinoschisis
Source: Front Med (Lausanne). 2025 Sep 30;12:1546953. doi: 10.3389/fmed.2025.1546953 (PMC12518284; doi:10.3389/fmed.2025.1546953)

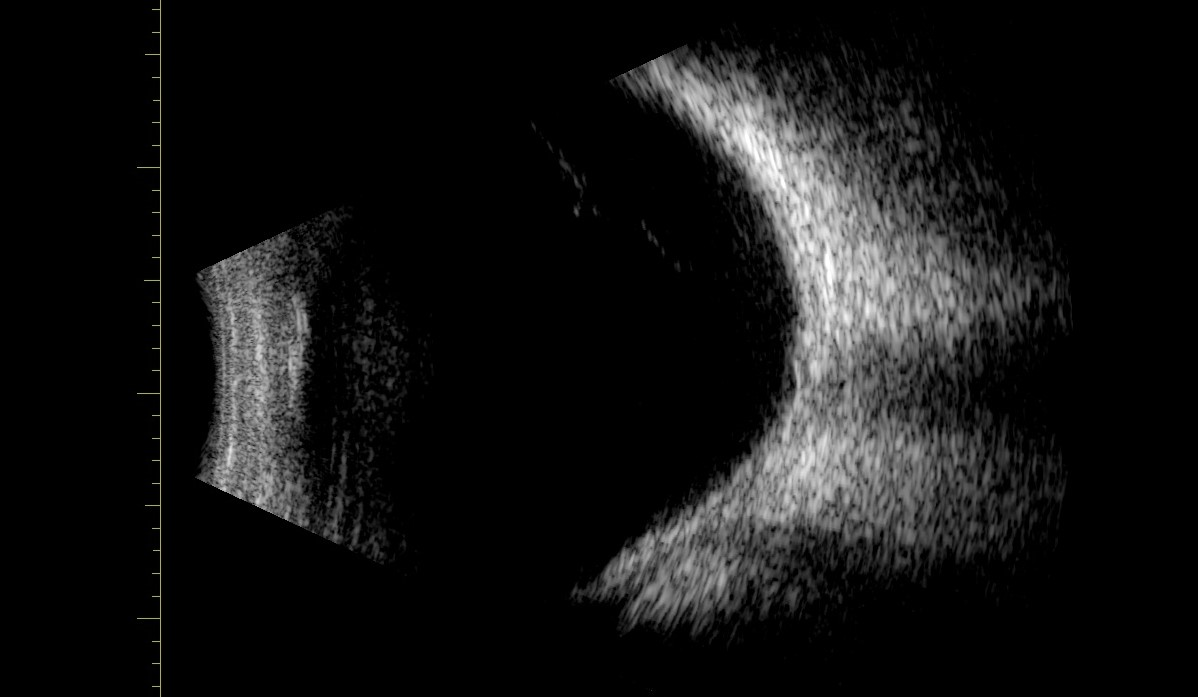

Supplement: SUPPLEMENTARY FIGURE 1 — B-ultrasonography revealed no vitreoretinal traction of the left eye. [file Image_1.TIF]

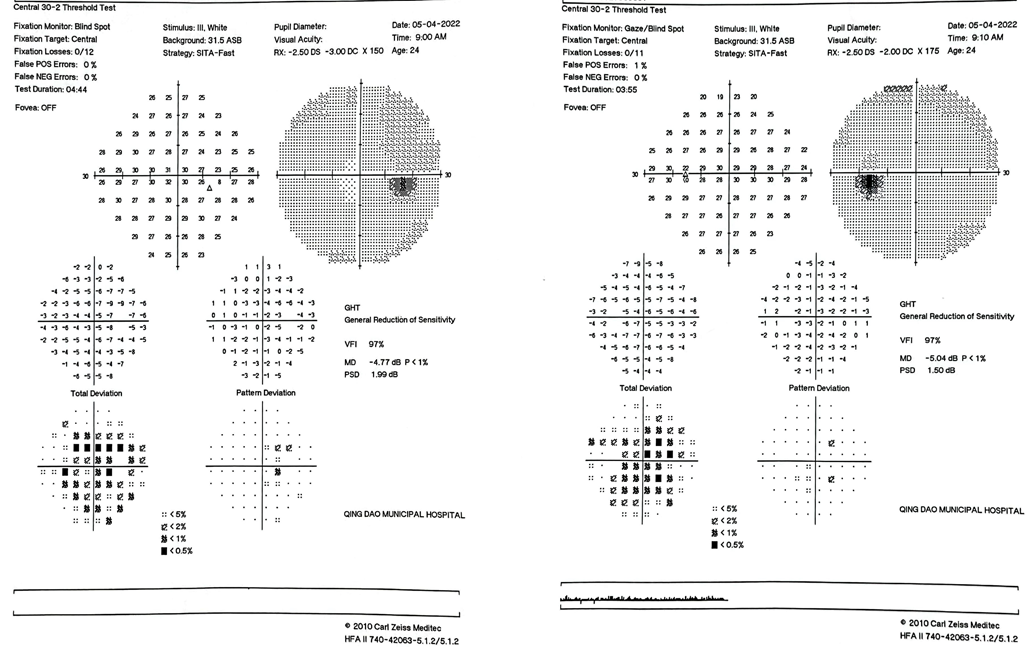

Supplement: SUPPLEMENTARY FIGURE 2 — Two weeks after the patient was diagnosed, although the OCT demonstrated that the retinoschisis was aggravated, there was no obvious abnormality in the binocular visual field. [file Image_2.TIF]
