# Supplementary material for: Case Report: A case of unexplained retinoschisis
Source: Front Med (Lausanne). 2025 Sep 30;12:1546953. doi: 10.3389/fmed.2025.1546953 (PMC12518284; doi:10.3389/fmed.2025.1546953)
Supplement: Supplementary file 3 [file Data_Sheet_1.PDF]

CARE Checklist of information to include when writing a case report

CARE case report guidelines

(cc) BY-NC-ND

Topic	Item	Checklist item description	Reported on Line
Title	1	The diagnosis or intervention of primary focus followed by the words "case report"	Page 1, title
Key Words	2	2 to 5 key words that identify diagnoses or interventions in this case report, including "case report"	Page 2/line 16
Abstract (no references)	3a	Introduction: What is unique about this case and what does it add to the scientific literature?	Page 2/line 4-6,13-15
	3b	Main symptoms and/or important clinical findings	Page 2/line 7-9
	3c	The main diagnoses, therapeutic interventions, and outcomes	Page 2/line 9-12
	3d	Conclusion—What is the main "take-away" lesson(s) from this case?	Page 2/line 13-15
Introduction	4	One or two paragraphs summarizing why this case is unique (**may include references**)	Page 2/line 19-29
Patient Information	5a	De-identified patient specific information	Page 3/line 32
	5b	Primary concerns and symptoms of the patient	Page 3/line 32-35
	5c	Medical, family, and psycho-social history including relevant genetic information	Page 3/line 35-42
	5d	Relevant past interventions with outcomes	Page 3-4/line 54-59, 60-63
Clinical Findings	6	Describe significant physical examination (PE) and important clinical findings	Page 3/line 43-59
Timeline	7	Historical and current information from this episode of care organized as a timeline	Not applicable
Diagnostic Assessment	8a	Diagnostic testing (such as PE, laboratory testing, imaging, surveys).	Page 3/line 48-53
	8b	Diagnostic challenges (such as access to testing, financial, or cultural)	Not applicable
	8c	Diagnosis (including other diagnoses considered)	Page 4/line 78-85
	8d	Prognosis (such as staging in oncology) where applicable	Page 4/line 68-74
Therapeutic Intervention	9a	Types of therapeutic intervention (such as pharmacologic, surgical, preventive, self-care)	Page 3-4/line 54-57, 63-64
	9b	Administration of therapeutic intervention (such as dosage, strength, duration)	Page 3/line 54-57
	9c	Changes in therapeutic intervention (with rationale)	Not applicable
Follow-up and Outcomes	10a	Clinician and patient-assessed outcomes (if available)	Page 4/line 70-72
	10b	Important follow-up diagnostic and other test results	Page 4/line 72-74
	10c	Intervention adherence and tolerability (How was this assessed?)	Not applicable
	10d	Adverse and unanticipated events	Not applicable
Discussion	11a	A scientific discussion of the strengths AND limitations associated with this case report	Not applicable
	11b	Discussion of the relevant medical literature **with references**	Page 7-8/line 173-220
	11c	The scientific rationale for any conclusions (including assessment of possible causes)	Page 6/line 143-144
	11d	The primary "take-away" lessons of this case report (without references) in a one paragraph conclusion	Page 6/line 144-148
Patient Perspective	12	The patient should share their perspective in one to two paragraphs on the treatment(s) they received	Not applicable
Informed Consent	13	Did the patient give informed consent? Please provide if requested	Yes ☑ No ☐
